# Trifluoperazine causes mast cell apoptosis through a secretory granule-mediated pathway

**DOI:** 10.1038/s41420-026-03122-x

**Published:** 2026-04-22

**Authors:** Marianthi Vraila, Jun Mei Hu Frisk, Animamalar Mayavannan, Mirjana Grujic, Erik Stigare, Adnan Lidian, Jenny Hallgren, Gunnar Pejler

**Affiliations:** 1 Department of Medical Biochemistry and Microbiology, Uppsala University, Uppsala, Sweden; 2 Department of Surgical Sciences, Uppsala University, Uppsala, Sweden

**Keywords:** Drug development, Preclinical research, Granulocytes, Cell death

## Abstract

Mast cells contribute to the pathology of various diseases, in particular allergic conditions. Therefore, it is essential to develop strategies that efficiently prevent their harmful effects under such circumstances. Here, we sought to evaluate the possibility of cell death induction as a potential means of selectively depleting mast cells. Previous work has suggested that mast cells are sensitive to regimes that target their acidic secretory granules, and the aim of this study was therefore to identify novel anti-mast cell compounds that act via a granule-mediated pathway. To this end, we evaluated trifluoperazine, an antipsychotic drug known to present lysosomotropic properties. We demonstrate that trifluoperazine is cytotoxic for mast cells, whereas multiple other cell types were resistant. Trifluoperazine induced mainly apoptotic cell death in mast cells. Further, our data indicate that trifluoperazine acts on mast cells by inducing secretory granule permeabilization. In support of this, trifluoperazine caused granule deacidification, accompanied by cytosolic acidification as well as translocation of tryptase from the secretory granules into the cytosol. Trifluoperazine-induced cell death and subsequent DNA degradation were profoundly abrogated when granule acidification was inhibited by the V-ATPase inhibitor bafilomycin A1, suggesting that the granule acidity has a key role in the cell death mechanism. Moreover, mast cell death in response to trifluoperazine was largely caspase-independent, whereas serine protease activity was shown to promote apoptosis-like vs. necrosis-like cell death. Overall, these findings introduce trifluoperazine as a novel anti-mast cell agent that induces cell death through granule permeabilization. Trifluoperazine may thus be evaluated for therapeutic intervention to ameliorate mast cell-mediated detrimental effects.

## Introduction

Mast cells (MCs) are multifaceted immune cells of the hematopoietic lineage involved in various homeostatic processes. However, MCs have also been implicated in numerous inflammatory diseases, in particular allergic conditions, as well as in additional pathologies such as atherosclerosis, arthritis, and cancer [1–4]. The most unique phenotypical feature of mature MCs is their high content of lysosome-like acidic granules, which are filled with several bioactive compounds. These compounds can be either preformed, such as histamine, lysosomal hydrolases, cytokines, and proteases, or newly synthesized in response to MC activation, with the latter including prostaglandins, leukotrienes, and growth factors [5, 6]. Upon appropriate activation, for example, through IgE receptor ligation, MCs degranulate and release their granule contents into the extracellular milieu. Thus, MC activation can induce a strong inflammatory response [5].

Given the implication of MCs in a wide range of disease conditions, the development of novel therapeutic approaches that limit their harmful actions has the potential to offer control over a plethora of pathological settings [7–10]. Notably though, currently available therapeutical options for intervening with MC actions, such as histamine receptor antagonists and MC stabilizers, aim to target only a limited fraction of MC mediators, providing some beneficial effects. However, it is known that MC-mediated diseases are mainly attributed to the synergistic effect of multiple proinflammatory mediators released by these cells [7, 11]. Therefore, an alternative strategy to effectively interfere with the MC-mediated effects could be to locally eliminate them through induction of selective apoptosis [as discussed in [12–14]].

Lysosomotropic agents are weakly basic amines that accumulate within acidic compartments such as lysosomes and MC secretory granules, and become trapped due to protonation. Once the concentration of such compounds exceeds a certain threshold, they exhibit detergent-like properties and can thereby permeabilize the lysosome/granule membranes. This can lead to cell death [15, 16]. In the present study, we aimed to investigate whether novel lysosomotropic agents that are compatible with human use could be employed to selectively deplete MC populations. This strategy was based on the notion that MCs have significantly higher abundance of cytosolic secretory granules than other immune cells. Moreover, the MC secretory granules contain cytotoxic substances that, once released into the cytosol, could potentially trigger the apoptotic machinery. Hence, we hypothesized that targeting of this unique feature of MCs might lead to selective cell death over other cell populations in the tissue [5, 17].

In this study, we evaluated trifluoperazine (‘Stelazine’) for this purpose. Trifluoperazine (TFP) is an approved anxiolytic and antipsychotic drug belonging to the phenothiazine family of drugs, and is used to treat schizophrenia and non-psychotic anxiety [18–20]. It has previously been shown that phenothiazines can possess lysosomotropic properties [21, 22], and we therefore reasoned that TFP might have the potential to cause selective MC death. Indeed, we show that TFP efficiently and selectively induces MC apoptosis, and that this occurs through a granule-mediated pathway.

## Results

### TFP shows cytotoxicity for MCs

**Fig. 1:**
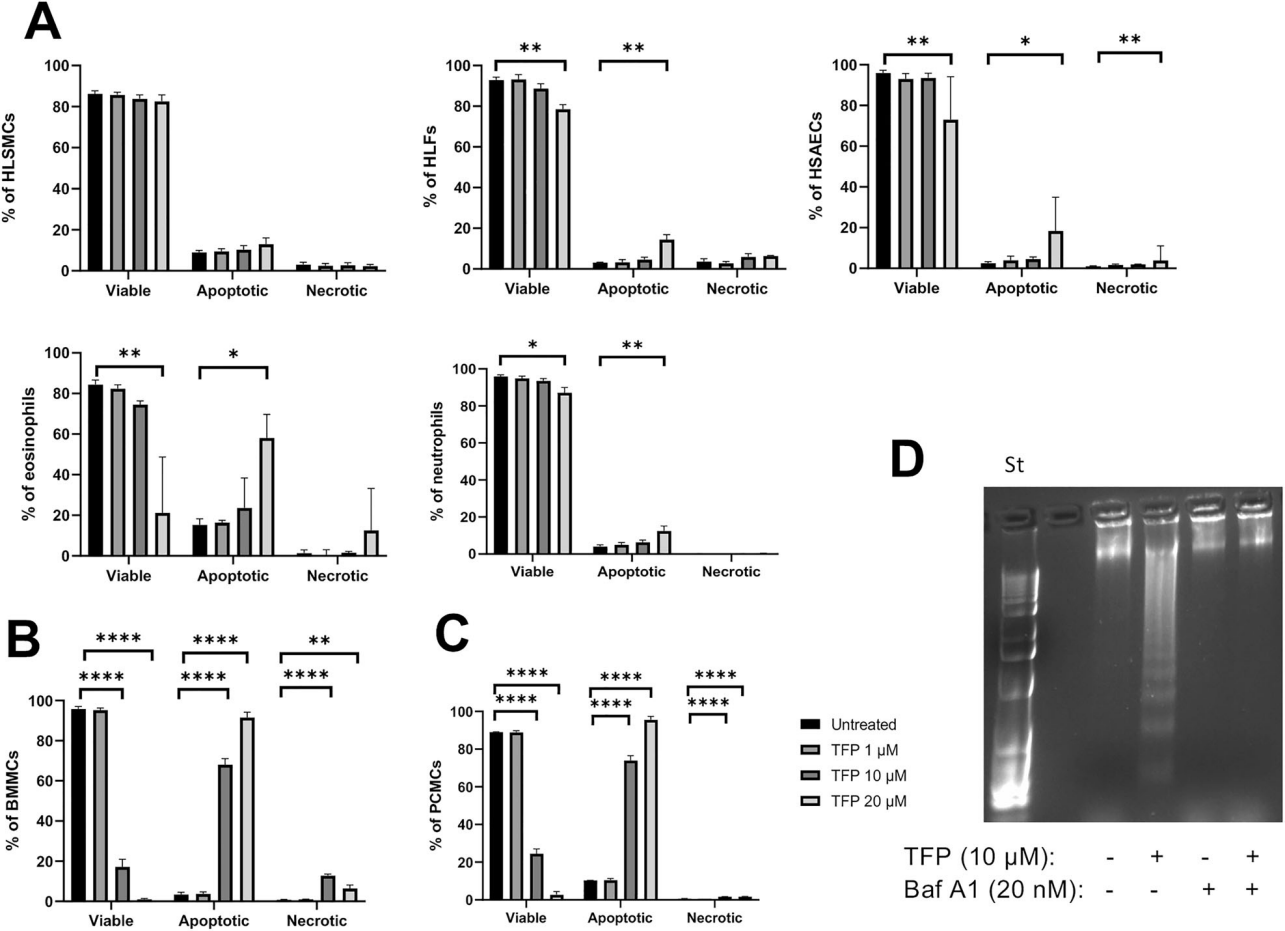
Trifluoperazine (TFP) at 10 μΜ shows selective cytotoxicity against MCs. **A** Human lung smooth muscle cells (HLSMCs), lung fibroblasts (HLFs), and small airway epithelial cells (HSAECs) were treated with TFP at the indicated concentrations for 24 h. Human peripheral blood eosinophils and neutrophils were treated with TFP for 2 h. Cell viability was assessed by staining the cells with Annexin V (AnnV) and DRAQ7. Viable cells, AnnV^−^ DRAQ7^−^; apoptotic cells, AnnV^+^ DRAQ7^−^; necrotic/late apoptotic cells, AnnV^+^ DRAQ7^+^. HSAECs, *n* = 6 from three independent experiments; HLSMCs, eosinophils, neutrophils, *n* = 4 from four independent experiments; HLFs, *n* = 3 from one individual experiment representative of three independent experiments (One-way ANOVA for HLSMCs, HLFs; Kruskal-Wallis for HSAECs; Friedman test for eosinophils, neutrophils). **B**–**C** Bone marrow-derived MCs (BMMCs) and peritoneal cell-derived MCs (PCMCs) treated under the same conditions as in (**A**) for 24 h. BMMCs, *n* = 5 from two independent experiments; PCMCs, n = 3 from one individual experiment representative of three independent experiments (One-way ANOVA). Untreated (control) cells were used for statistical comparisons to all other groups in all figures. The bar charts show mean values + SEM or median + interquartile range. *P < 0.05; **P < 0 .01; ****P < 0.0001. **D** Effect of TFP on DNA degradation. MCs were preincubated with bafilomycin A1 (Baf A1) (20 nM) for 2 h followed by treatment with TFP (10 μΜ) for 2 h. DNA was extracted from MCs and fragmentation was assessed by agarose gel electrophoresis. St standard marker.

We first assessed the effect of trifluoperazine (TFP) on the viability of a panel of primary human cells. To this end, airway smooth muscle cells (SMCs), fibroblasts (HLFs), epithelial cells (HSAECs), blood eosinophils and neutrophils were treated with TFP at different concentrations (0–20 μΜ) for 24 h, followed by Annexin V/DRAQ7 staining to monitor the fraction of viable (Annexin V^-^ DRAQ7^-^), apoptotic (Annexin V^+^ DRAQ7^-^) and necrotic/late stage apoptotic (Annexin V^+^ DRAQ7^+^) cells. These assessments revealed that the airway smooth muscle cells, fibroblasts, epithelial cells, and neutrophils were all essentially resistant to TFP, with only modest cytotoxicity observed at the highest TFP concentration used (20 µM) (Fig. 1A). On the contrary, eosinophils were slightly sensitive, with a significant reduction of viability seen at 20 µM of TFP, whereas no significant cytotoxicity was observed at 10 µM or lower concentrations of the drug (Fig. 1A; Fig. S5).

To examine the effect of TFP on MCs, the drug was administered to mouse bone marrow-derived MCs (BMMCs) and viability was assessed after 24 h. As seen in Fig. 1B, TFP was highly cytotoxic for the BMMCs, with ~80% loss of viability seen already at 10 µM TFP and with no remaining viable cells observed at 20 µM of the drug. BMMCs are primary cells, being prominent tools for studies of MC function, but it is notable that this MC population does not represent fully mature MCs and has a somewhat lower content of secretory granules as compared with fully mature MCs found in vivo [23]. To assess whether TFP is also cytotoxic for fully mature MCs, we evaluated its effects on peritoneal cell-derived MCs (PCMCs). Indeed, TFP was highly cytotoxic also for PCMCs, thus suggesting that TFP has cytotoxic effects on multiple MC populations (Fig. 1C).

To further evaluate whether these observations generalize to human MCs, mixed cell populations derived from nasal polyps as well as a MC line (LUVA) were treated with TFP at various concentrations. TFP at 10 μΜ caused a trend towards reduction of MC frequency in dispersed cells from human nasal polyps (Fig. S3B; gating strategy shown in Fig. S3A). In contrast, no significant effects on T- & B lymphocytes (Fig. S3C) or CD14^+^ monocytes (Fig. S3D) were seen in response to TFP. Furthermore, TFP was not cytotoxic for LUVA cells, showing only minor cytotoxicity at 20 μΜ (Fig. S3Ε).

**Fig. 2:**
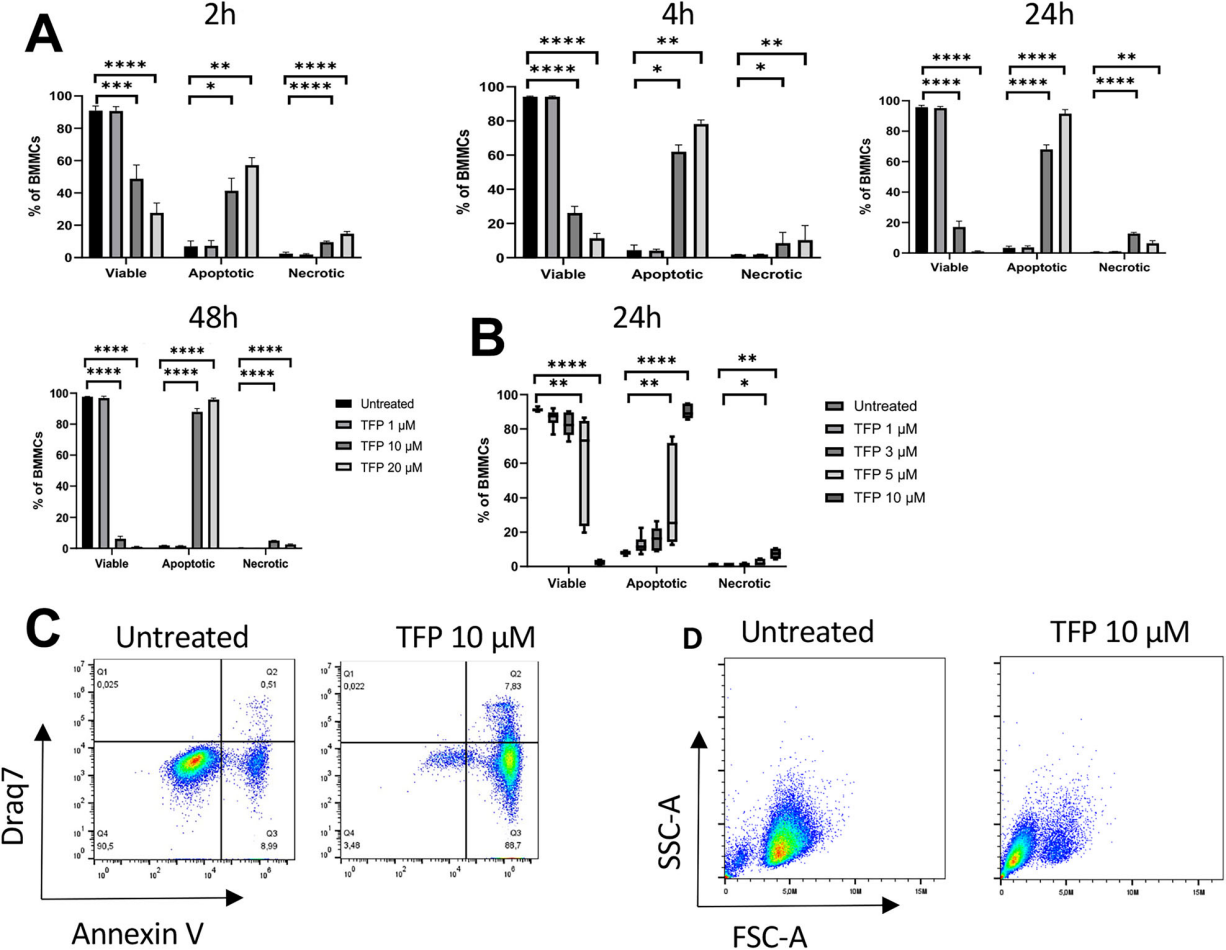
Trifluoperazine (TFP) induces apoptotic cell death in MCs. **A** Time-course induction of cell death in BMMCs. Cells were incubated with TFP at the indicated concentrations for 2 h, 4 h, 24 h and 48 h, respectively, followed by staining with Annexin V (AnnV) and DRAQ7. Viable cells, AnnV^−^ DRAQ7^−^; apoptotic cells, AnnV^+^ DRAQ7^−^; necrotic/late apoptotic cells, AnnV^+^ DRAQ7^+^. *n* = 5 from two independent experiments (One-way ANOVA for 2, 24 h, 48 h; Kruskal-Wallis for 4 h). **B** Dose-response of TFP-induced cell death at 24 h. Cell viability was assessed by staining the cells with AnnV and DRAQ7. *n* = 6 from three independent experiments (Kruskal-Wallis). Untreated (control) cells were used for statistical comparisons to all other groups in all graphs. The bar charts show mean values + SEM or median+ interquartile range. The box and Whisker plots show median values, interquartile range, and minimum to maximum values. *P < 0.05; **P < 0.01; ***P < 0.001; ****P < 0.0001. **C** Representative flow plots depicting AnnV/DRAQ7 staining of untreated and TFP-treated BMMCs after 24 h. **D** Flow plots showing forward scatter area (*FSC-A*) and side scatter area (*SSC-A*) of untreated and TFP-treated BMMCs after 24 h.

As seen in Fig. 2A–C (gating strategy shown in Fig. 2C), TFP-sensitive MCs were predominantly Annexin V-positive and DRAQ7-negative, indicating that TFP predominantly causes cell death with apoptosis-like characteristics rather than necrosis-like cell death. However, Annexin V/DRAQ7 double positive cells could also be seen to a more limited extent, indicating necrosis or late-stage apoptosis. Moreover, extensive DNA fragmentation was seen in TFP-treated MCs (Fig. 1D). Since DNA fragmentation is a hallmark feature of apoptosis [24], these findings reinforce that TFP acts on MCs via induction of apoptotic cell death. Notably, also TFP-sensitive eosinophils mainly underwent apoptosis-like cell death in response to TFP (Fig. 1A).

A time course assessment revealed that pronounced MC death was seen starting from ~2 h of incubation with TFP, and that a progressive increase in the fraction of non-viable cells was seen when extending the incubation time up to 48 h (Fig. 2A). Further, a dose response assessment, where TFP concentrations below 10 µM were included, showed that significant cell death was observed starting from 5 µM of TFP (Fig. 2B). Notably, TFP-sensitive MCs were almost exclusively Annexin V^+^/DRAQ7^-^, across multiple incubation times and across multiple TFP concentrations, reinforcing that TFP overall causes MC death with apoptosis-like characteristics. Apoptosis-like cell death was also supported by flow cytometry analysis, revealing decreased cellular size of the MCs in response to TFP (Fig. 2D).

### TFP causes granule permeabilization in MCs

**Fig. 3:**
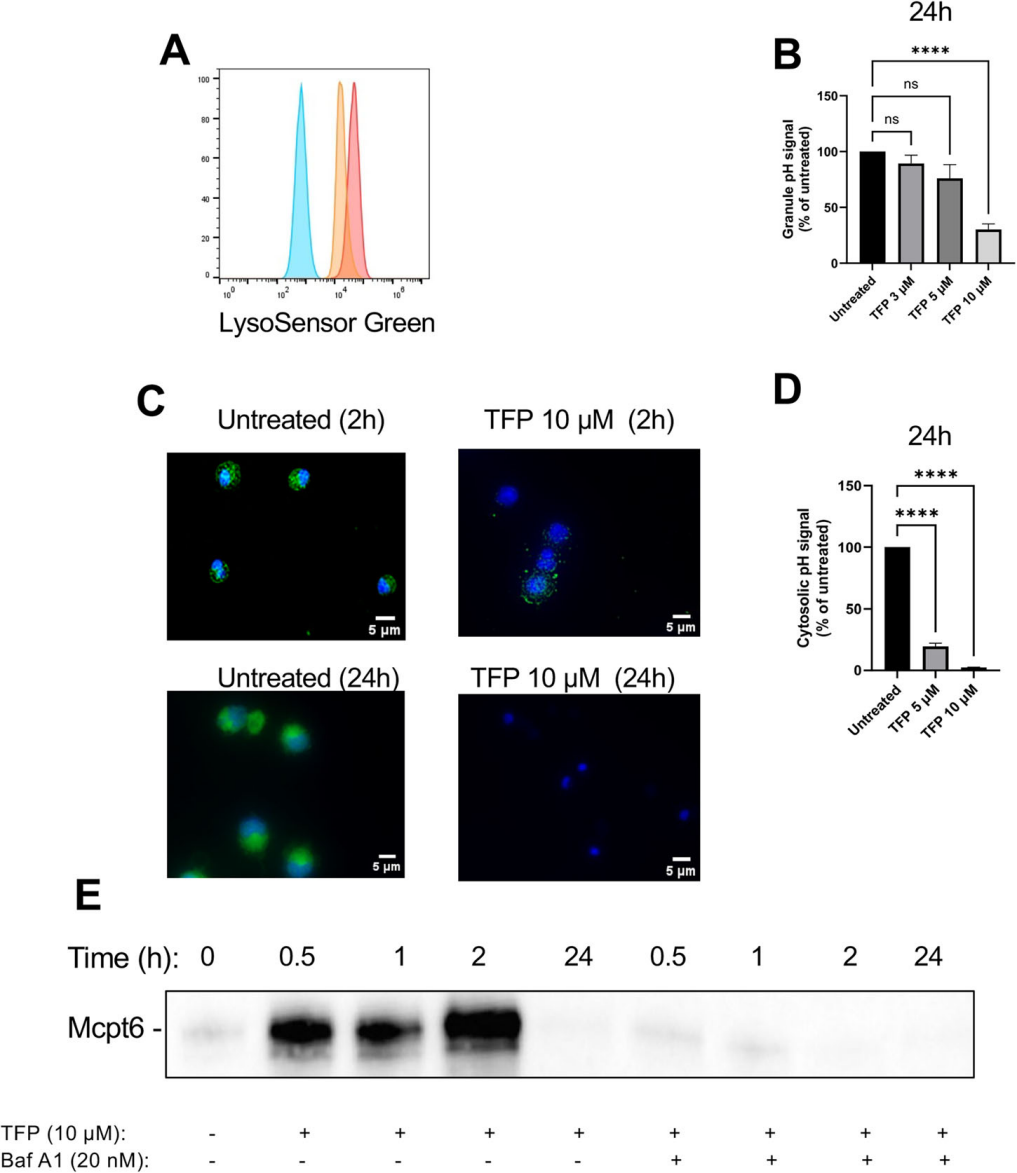
Trifluoperazine (TFP) causes granule deacidification and a decrease in cytosolic pH. **A** Representative flow plot and **B** quantification of LysoSensor Green expression as mean fluorescence intensity (MFI) with (orange) or without TFP (10 μM) (red) versus unstained control cells (blue) after 24 h incubation. *n* = 6 from three independent experiments (One-way ANOVA). ****P < 0.0001; ns, non-significant. **C** BMMCs were incubated in the presence or absence of TFP at the indicated concentrations for 2 h or 24 h, followed by staining with LysoSensor Green (1 μM) for 30 min and imaging using fluorescence microscopy, after nuclear staining with DAPI (blue). Note the reduction of LysoSensor intensity (green) after treatment with TFP, indicating increase of granular acidity. **D** Similar setups as in (**B**) but cells were stained with BCFL-AM for 30 min and cytosolic pH was assessed as MFI. *n* = 4 from two independent experiments (One-way ANOVA). ****P < 0.0001. Untreated (control) cells were used for statistical comparisons to all other groups in all graphs. The bar charts show mean values + SEM. **E** Representative Western blot analysis of Mcpt6 in cytosolic extracts. BMMCs were incubated in the presence or absence of bafilomycin A1 (Baf A1) (20 nM) for 4 h followed by treatment with TFP (10 μΜ) for 20 h. Cells treated with PBS or TFP alone were used as controls. Cytosolic extracts were prepared at the indicated time points.

Phenothiazines such as TFP are known to have lysosomotropic properties [21, 22], and we thus hypothesized that the cytotoxic effects of TFP on MCs involves permeabilization of their secretory granules, considering that these represent acidic compartments with lysosome-like properties [25]. To evaluate this possibility, we first incubated untreated and TFP-treated MCs with LysoSensor green (LSG). LSG is a probe that localizes to acidic compartments, such as secretory granules, and produces strong fluorescence under acidic conditions. In agreement with their high content of secretory granules, untreated MCs were stained strongly with LSG (Fig. 3A). As depicted in Fig. 3B, treatment of the MCs with TFP caused a dose-dependent decrease in LSG fluorescence, indicating an increase in intragranular pH (granule deacidification). These findings were also supported by fluorescence microscopy analysis, demonstrating pronounced LSG staining of untreated MCs, whereas markedly weaker LSG staining was seen after treatment of the cells with TFP (Fig. 3C). These findings are thus in agreement with a mechanism involving granule permeabilization. Conceivably, such a mechanism would lead to efflux of acidic content from the granules into the cytosol. To further evaluate this possibility, we also stained the cells with BCFL-AM, a probe that is used for measurements of cytosolic pH. Indeed, TFP induced a pronounced, dose-dependent reduction in the fluorescence of the cytosolic probe, indicating a decrease in cytosolic pH (Fig. 3D).

To provide further support for a granule-permeabilizing mechanism, we prepared cytosolic extracts from untreated and TFP-treated MCs and assessed whether TFP caused translocation of tryptase (Mcpt6), a major granule component of MCs, from the granules to the cytosolic compartment. Indeed, TFP mediated release of tryptase into the cytosol (Fig. 3E; see Fig. S4 for loading control). Together, these data suggest that TFP acts on MCs by permeabilizing their secretory granules, leading to efflux of protons and other components from the granules to the cytosol.

### Blockade of granule acidification abrogates the cytotoxic effect of TFP on MCs

**Fig. 4:**
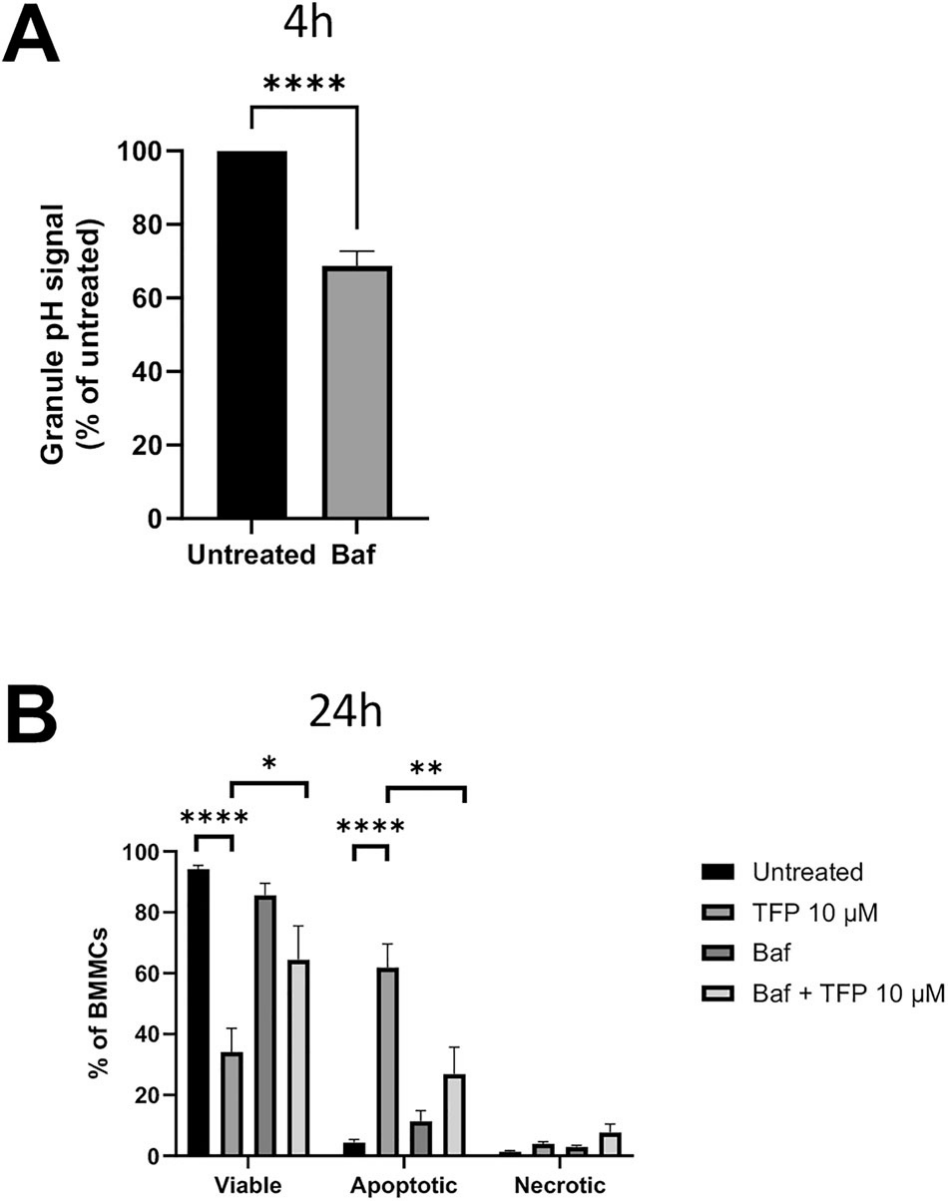
Loss of granule acidity reduces the effect of trifluoperazine (TFP) on MC apoptosis. **A** BMMCs were treated with bafilomycin A1 (Baf A1) (20 nM) for 4 h followed by staining with LysoSensor Green (1 μM) for 30 min to assess granule acidity. LysoSensor Green expression is given as mean fluorescence intensity (MFI). *n* = 5 from two independent experiments (two-tailed unpaired *t* test). **B** BMMCs were preincubated in the presence or absence of bafilomycin A1 (20 nM) followed by treatment with TFP (10 μM) for 24 h. Cell viability was assessed by staining with Annexin V (AnnV) and DRAQ7. Viable cells, AnnV^−^ DRAQ7^−^; apoptotic cells, Ann V^+^ DRAQ7^−^; necrotic/late apoptotic cells, AnnV^+^ DRAQ7^+^. *n* = 8 from three independent experiments (One-way ANOVA). Untreated (control) or TFP-treated MCs were used for statistical comparisons to all other groups in (**A**) and (**B**), respectively. The bar charts show mean values + SEM. *P < 0.05; **P < 0.01; ****P < 0.0001.

MC granules have an acidic pH (5.2–6.1) [26–28] and previous studies have shown that the acidification of the MC granules is dependent on vacuolar ATPase (v-ATPase) [29, 30]. Having shown that TFP causes efflux of protons to the cytosol leading to decreased cytosolic pH, we next considered the possibility that such pH effects could drive the responsiveness of MCs to TFP. To test this, we assessed whether interference with granule acidification influences the efficacy of TFP in inducing MC apoptosis. For this purpose, we subjected the cells to bafilomycin A1, a specific V-ATPase inhibitor. As expected, treatment of the MCs with bafilomycin A1 caused a marked reduction in granule acidity (Fig. 4A). Further, bafilomycin A1 treatment resulted in significant protection of the MCs against the cytotoxic effects of TFP (Fig. 4B), and it was also observed that the DNA fragmentation induced by TFP was completely abrogated when the cells had been incubated with bafilomycin A1 (Fig. 1D). Moreover, the TFP-induced translocation of Mcpt6 into the cytosol was blocked by treatment of the MCs with bafilomycin A1 (Fig. 3E). Hence, these data indicate that granule acidity is essential for the ability of TFP to execute secretory granule permeabilization leading to MC apoptosis.

### TFP-induced cell death is largely caspase-independent

**Fig. 5:**
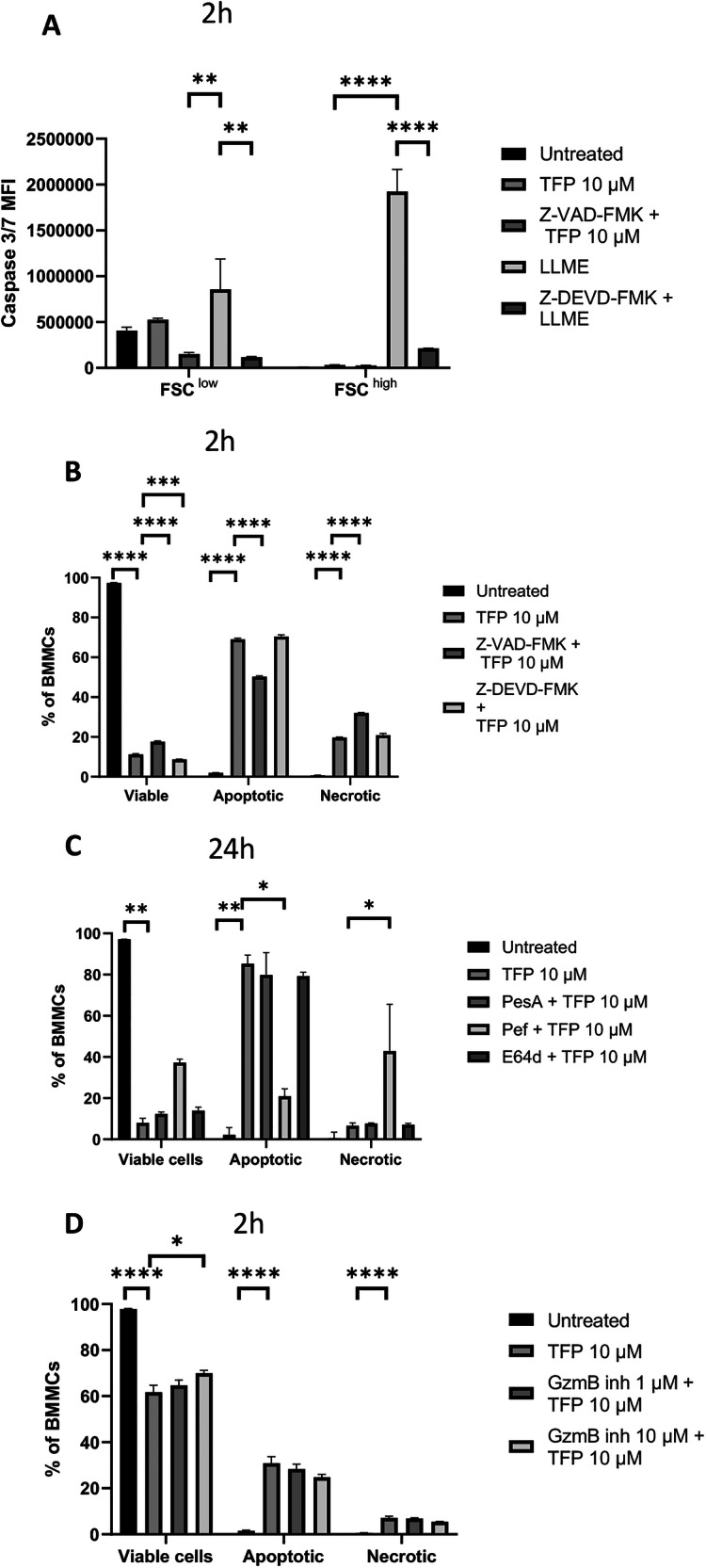
Trifluoperazine (TFP)-induced cell death in MCs is caspase-independent. **A** BMMCs were preincubated with the pan-caspase inhibitor Z-VAD-FMK (20 μΜ) for 30 min followed by 2 h incubation with TFP (10 μΜ). BMMCs treated with LLME (500 μΜ) for 1 h were included as positive control. To assess the activity of the caspase 3/7 inhibitor Z-DEVD-FMK, BMMCs were preincubated with Z-DEVD-FMK (20 μΜ) for 30 min followed by incubation with LLME (500 μΜ) for 1 h. Caspase 3/7-activity is given as mean fluorescence intensity (MFI) of the caspase 3/7 green detection agent. *n* = 3 from three independent experiments (Two-way ANOVA). TFP- and LLME-treated cells were used for statistical comparisons to all other groups. Data for gated FSC^low^ and FSC^high^ cells are displayed for comparison. **B** BMMCs were preincubated with Z-VAD-FMK (20 μΜ) and/or or caspase 3/7 inhibitor Z-DEVD-FMK (20 μΜ) for 30 min, followed by 2 h incubation with TFP (10 μΜ). Cell viability was assessed by staining the cells with Annexin V (AnnV) and DRAQ7. Viable cells, AnnV^−^ DRAQ7^−^; apoptotic cells, Ann V^+^ DRAQ7^−^; necrotic/late apoptotic cells, AnnV^+^ DRAQ7^+^. *n* = 3 from one individual experiment, representative of three independent experiments (One-way ANOVA). **C** BMMCs were preincubated with pepstatin A (PesA) (50 μΜ), Pefabloc SC (Pef) (0.1 mM) or E64d (20 μΜ) for 30 min, followed by 24 h incubation with TFP (10 μΜ). Cell viability was assessed by staining with AnnV and DRAQ7. *n* = 4 from two independent experiments (Kruskal-Wallis). **D** Similar setups as in (**B**) but cells were preincubated with the granzyme B (GzmB) inhibitor Z-AAD-CMK (1 μΜ, 10 μΜ) before treatment with TFP. *n* = 3 from one individual experiment, representative of two independent experiments (One-way ANOVA). TFP-treated cells were used for statistical comparisons to all other groups in figures (**B**–**D**). The bar charts show mean values + SEM or median+ interquartile range. ∗*P* < 0.05; ∗∗*P* < 0.01; ∗∗∗*P* < 0.001; ∗∗∗∗*P* < 0.0001.

To further elucidate the mechanistic basis for the TFP-induced MC death, we first analysed the contribution of caspase activation. TFP induced only modest caspase 3/7 activation in both FSC^low^ and FSC^high^ MC populations, and this activity was inhibited by the pan-caspase inhibitor Z-VAD-FMK (Fig. 5A). More robust caspase 3/7 activation was seen in response to LLME, a compound that previously has been shown to induce caspase 3/7 activation in MCs [31], hence serving as a positive control for caspase 3/7 activity. In response to Z-DEVD-FMK, the LLME-induced caspase 3/7 activity was blocked (Fig. 5A). Further, preincubation of cells with Z-VAD-FMK caused only a slight reduction of the apoptosis-like cell death in response to TFP. It was also noted that treatment of the MCs with Z-VAD-FMK caused a slight shift in the mode of cell death in response to TFP, from mainly apoptotic cell death to necrosis/late-stage apoptosis (Fig. 5B). In contrast to Z-VAD-FMK, the selective caspase 3 inhibitor (Z-DEVD-FMK) did not provide any protection of the MCs against the cytotoxic effects of TFP (Fig. 5B). Together, these findings indicate that caspase activation has only a minor role in TFP-induced MC death. We also evaluated the contribution of the pro-apoptotic serine protease granzyme B (GzmB). Previous studies have shown that GzmB is expressed by MCs and contributes to cell death upon treatment with lysosomotropic agents [32]. However, only low levels of GzmB activity were detected in TFP-treated MCs and the GzmB inhibitor Z-AAD-CMK only slightly reduced the extent TFP-induced cell death (Fig. 5D; see Fig. S1C for positive controls revealing that the inhibitor active). This indicates that GzmB is not a major inducer of apoptosis in MCs upon treatment with TFP.

Since the data above show that caspases and granzyme B have only a minor role in executing MC death in response to TFP, we reasoned that other proteolytic enzyme might contribute. To assess this, we treated MCs with general inhibitors of either aspartic acid (pepstatin A; see Fig. S1B for positive controls revealing that the inhibitor active), serine (Pefabloc SC), or cysteine (E64d; see Fig. S1A for positive controls revealing that the inhibitor active) proteases prior to TFP treatment. As shown in Fig. 5C, preincubation of the cells with either pepstatin A or E64d did not rescue MCs from cell death. In contrast, Pefabloc SC induced a trend towards a reduction in TFP-induced cell death (Fig. 5C). More strikingly, serine protease inhibition caused a dramatic shift from apoptosis-like cell death to necrosis/late-stage apoptosis in response to TFP (Fig. 5C), hence suggesting that serine protease activity other than granzyme B has an important role in regulating the mechanism of cell death in TFP-treated MCs.

### TFP causes granule distortion in MCs

**Fig. 6:**
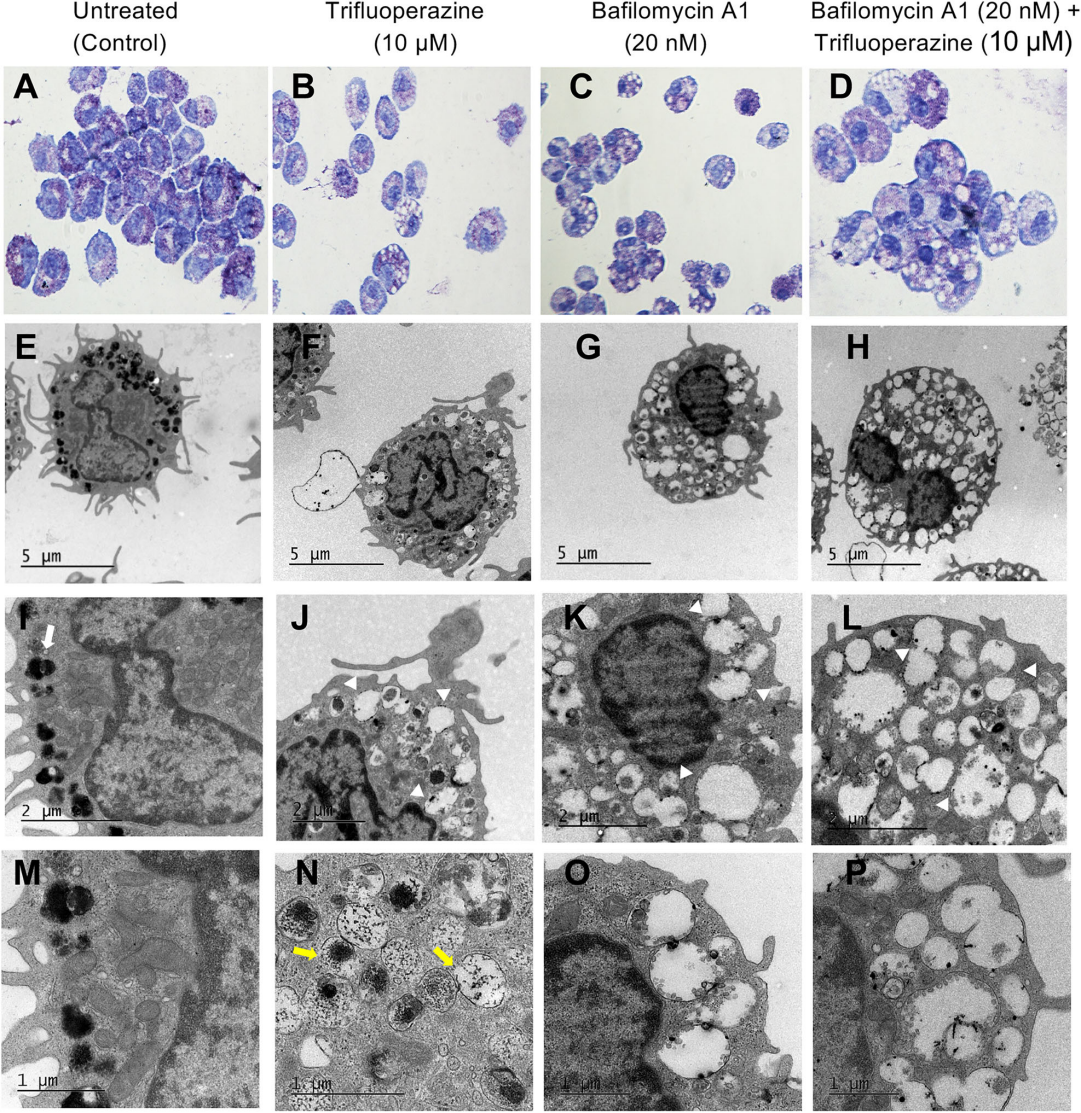
Trifluoperazine (TFP) causes distortion of granule morphology in MCs. BMMCs were incubated with or without TFP (10 μM) in the presence or absence of bafilomycin A1 (20 nM) for 24 h. **A**–**D** Cytospin slides were prepared and stained with toluidine blue. **E**–**H** Transmission electron microscopy (TEM) analysis of MCs treated as indicated above. **I**–**P** Enlarged images indicating the distortion of secretory granules; The white arrow in (**I**) indicates the electron dense granule core surrounded by electron lucent content; the white arrowheads in (**J**, **K**, **L**) highlight the distorted granule morphology observed in response to TFP. The yellow arrows in (**N**) indicate pore formation in granule membranes after treatment with TFP; note that such pores were not visible after treatment of the MCs with bafilomycin A1 (**O**, **P**).

Next, we investigated whether TFP affects the morphology of MCs, with a specific focus on secretory granule integrity. To this end, MCs were incubated ± TFP, either in the absence or presence of bafilomycin A1, followed by centrifugation onto cytospin slides and toluidine blue staining. As seen in Fig. 6A–D, light microscopic analysis revealed that TFP caused pronounced morphological changes of the MCs, as manifested by membrane blebbing, swelling of granules, and apparent loss of granule content. To provide a more detailed insight into how TFP affects MC morphology, we conducted transmission electron microscopy (TEM) analysis. This showed, as expected, that untreated MCs had a high content of secretory granules. These presented the typical ultrastructure, consisting of electron-dense crystalline cores surrounded by electron-translucent matrix (Fig. 6E, I). After treatment of the MCs with TFP, marked swelling of the granules was noted (Fig. 6F, J). This was accompanied by a dramatic reduction in granule content, clearly in line with efflux of granule content into the cytosol. In line with this, it was noted that the granule membranes of TFP-treated cells were partly discontinuous, i.e., in agreement with granule permeabilization (Fig. 6N). Moreover, the TEM analysis provided further insight into the morphological effects of TFP on MCs, revealing alterations in cell morphology indicative of apoptosis-like cell death, including membrane blebbing (Fig. 6F, J). It was also notable that the cell membranes were largely intact, this being in agreement with apoptotic cell death rather than necrosis (Fig. 6F, J). In agreement with our previous studies [30], treatment of the MCs with bafilomycin A1 caused considerable swelling of the granules into a vacuole-like morphology (Fig. 6G, K, O), most likely due to effects of increased granule pH on the organization and electrostatic interactions between the granule compounds [30]. Notably, bafilomycin A1 protected the cells from the cytotoxic effects of TFP, preventing the membrane blebbing (Fig. 6H, L). It was also observed that the granule content was to some extent accumulated close to or at the border of the granule membranes in TFP-treated cells that also received bafilomycin A1 (Fig. 6L, P). The latter is compatible with a scenario in which bafilomycin A1 blocks granule permeabilization, and that granule content is thereby retained within the granules, being localized close to the granule membranes.

## Discussion

MCs are known to contribute to the pathophysiology of allergic diseases such as asthma, as well as malignancies and autoimmune diseases [4]. MCs are therefore emerging as potential therapeutic targets in numerous pathological settings [17]. In this study we show that TFP represents a novel anti-MC agent, with potent cytotoxicity against purified MC populations. Thereby, TFP may emerge as a novel therapeutic agent with the potential of suppressing symptoms caused by activated MCs in pathological settings where MCs have a detrimental impact.

To assess the selectivity of TFP for MCs vs. other cell types, we employed a panel of cell populations including primary human lung fibroblasts, smooth muscle cells, epithelial cells, neutrophils, eosinophils as well as the immortalized human mast cell line (LUVA), and investigated their sensitivity to TFP. Our data revealed that the viability of these cells (except for eosinophils; see above) was not affected by TFP at concentrations that were cytotoxic for both mouse and human MCs. Further, treatment with TFP did not affect the frequency of either nasal polyp CD14^+^ monocytes or T- & B lymphocytes. Importantly, TFP was cytotoxic for MCs at 10 μM, which corresponds to clinically relevant doses (0.4–5.6 ng/ml plasma) [33, 34]. It is also important to note that TFP, at this concentration, appeared to be cytotoxic for human nasal polyp MCs, although a high sample-to-sample variation was seen in these cell populations.

It is notable that human blood eosinophils were sensitive to TFP, although higher concentrations of TFP were required to cause cell death in eosinophils vs. MCs. The cytotoxic effect of TFP on eosinophils could potentially be explained by the fact that eosinophils, similar to MCs, have high numbers of lysosome-like acidic granules [35]. Of note, in comparison with eosinophils, neutrophils have lower amounts of granules and these are less acidic [36], which may explain why neutrophils were not sensitive to TFP. Our data reveal that LUVA cells were resistant to TFP, which is in line with our previous observation that this cell line is refractory to other types of lysosomotropic agents [29]. It is not yet clear why the LUVA cells are resistant to TFP and other lysosomotropic agents. However, likely explanations might include differing granule density, less content of granule-contained cytotoxic compounds, a larger nuclear:cytoplasmic ratio, and/or less granule acidity in comparison with primary MCs [37].

TFP is an established drug that is primarily approved for the treatment of psychiatric diseases [18–20]. Therefore, this study uncovered a novel use of this drug as an agent capable of selectively depleting MCs. Notably, human lung fibroblasts, smooth muscle cells and epithelial cells are altogether known to resemble the three layers of the airway wall. Thus, TFP at 10 μΜ may provide a favorable therapeutic window that would enable its use in MC-associated diseases like asthma without producing severe adverse effects on healthy tissue. This notion is further supported by previous studies demonstrating that 24 h incubation of normal lymphocytes with phenothiazines including TFP, up to 10 μΜ, does not affect cell viability [38]. Our preliminary data further suggest the beneficial use of TFP in depleting MCs in the nasal polyps.

Our data strongly indicated that TFP induces cell death through an apoptosis-like mechanism, as revealed by Annexin V/DRAQ7 staining. This was also supported by TEM analysis, where we demonstrated signs of apoptosis-like cell death including membrane blebbing, while the outer cell membrane was intact. Furthermore, the potential of TFP to induce apoptotic cell death increased over prolonged incubation times (up to 48 h), with no substantial induction of necrosis. This finding is of considerable importance in a clinical context since apoptosis is more preferable when compared to necrosis, because the former is a non-inflammatory mode of cell death while necrosis results in inflammation and damage of the surrounding tissues [39, 40].

Mechanistically, we show that TFP caused permeabilization of the MC secretory granules, as indicated by an increase in intragranular pH and a subsequent drop in cytosolic pH. Interestingly, although TFP, both at 5 and 10 μΜ significantly reduced the cytosolic pH, only TFP at 10 μΜ caused a profound raise in the granule pH. This suggests that the higher TFP concentration is more efficient in inducing leakage of protons and proteases from permeabilized granules into the cytosol. A striking observation was that the release of granule-localized tryptase, following granule permeabilization, was detected at time points when loss of viability was not substantial. This suggests that granule permeabilization is the main driving factor of TFP-induced apoptosis, rather than a secondary effect of cell death. Notably, tryptase was not detected in the cytosolic extracts after treatment with the drug for 24 h. We are not able to explain this finding with certainty, although a plausible explanation could be that tryptase is released from the cells after prolonged exposure to TFP, possibly due to compromised cell membrane integrity. Alternatively, the prolonged exposure of tryptase to elevated pH is known to induce monomerisation of the tryptase tetramer [41], which can lead to autoproteolysis [30].

In further agreement with a granule-mediated pathway, the TEM analysis confirmed that the secretory granule integrity was substantially compromised in response to TFP. Importantly, our data show that the granule content was significantly reduced after treatment with TFP. Based on these findings, we reasoned that the cytosolic acidification that follows granule permeabilization in response to treatment with TFP may have a role in the induction of cell death. In concordance with this notion, impairment of granule acidification by bafilomycin A1 profoundly inhibited the ability of TFP to induce apoptosis. Altogether, according to recent nomenclatures of cell death mechanisms [42], our findings indicate that TFP induces lysosome-dependent cell death in MCs.

The mechanism by which the cytosolic acidification contributes to induction of cell death in MCs is intriguing. There is evidence suggesting that certain DNAses, including DNAse II, are activated when the intracellular pH drops and mediate DNA degradation, a well-known feature of apoptosis [43–46]. One possible scenario could thus be that cytosolic acidification following granule permeabilization can lead to the activation of such DNAses, and that this could contribute to TFP-induced cell death. In agreement with this possibility, we demonstrated that TFP induced significant DNA degradation at a time point where cell death was pronounced. In further agreement with such a scenario, blockade of granule acidification efficiently inhibited DNA degradation in response to TFP. Interestingly, a similar mechanism has been proposed to explain the effects of phenothiazines on leukemic cells [38].

Cytosolic acidification is known to be a common feature of apoptotic cell death and could be either caspase-dependent or independent [47]. Since TFP-treated MCs show signs of apoptosis, we assumed that caspases, in particular effector caspases 3/7, may have an impact on cell death. However, TFP did not cause a significant increase in caspase 3/7 activation, and caspase inhibition by either the selective caspase-3 inhibitor Z-DEVD-FMK or the pan-caspase inhibitor Z-VAD-FMK, had only minor effects on cell viability after TFP treatment. Hence, this indicates that caspases do not contribute substantially to the execution of cell death in TFP-treated MCs.

As an alternative mechanism of cell death in response to TFP we considered that granule damage would release proteases stored in MC secretory granules including cysteine cathepsins, aspartic acid or serine proteases into the cytosol. Given the implication of such proteases to certain types of cell death [15, 48], it is plausible that they could proteolytically activate pro-apoptotic compounds, leading to apoptosis. However, inhibition of either of these protease classes did not significantly prevent apoptosis in response to TFP, arguing against a major role of granule-localized proteases in the execution of cell death. Notably though, although serine protease blockade did not rescue MCs from cell death, we observed that inhibition of this protease class led to a profound shift from cell death with apoptosis-like to necrosis-like characteristics. A plausible explanation for this could be that serine proteases escaping from the granules into the cytosol act on cytosolic substrates such that cell death is executed by an apoptotic- rather than necrotic pathway. However, further investigations are required to define the exact contribution of serine protease activity to the regulation of MC death in response to TFP.

Taken together, the present study introduces the repurposing of TFP as a novel anti-MC agent, which induces MC cell death and DNA degradation through a granule-mediated pathway. This introduces the possibility of using TFP or other lysosomotropic agents as a means to selectively deplete MCs in clinically relevant settings. Potentially, TFP may thus become further evaluated for therapeutic intervention in pathological settings in which MCs contribute.

## Materials and methods

Trifluoperazine dihydrochloride (TFP), Pefabloc SC, pepstatin A, Z-AAD-CMK and E64d were purchased from Sigma-Aldrich. H-Leu-Leu-OMe (LLME) was purchased from Bachem. Bafilomycin A1 was obtained from Invivogen. Z-DEVD-FMK and Z-VAD-CMK were obtained from AH Diagnostics.

### Mice

Mice were on C57BL/6 J genetic background. Both female and male mice (6–10 weeks of age) were used. The animal experiments were conducted in compliance with ethical regulations, and were approved by the local ethical committee (Uppsala djurförsöksetiska nämnd, Uppsala, Sweden; no C 31/14). Randomisation or blinding was not used.

### Cell culture

Mouse bone marrow-derived MCs (BMMCs) were generated and cultured according to an earlier described protocol [49] with minor modifications. Briefly, cells were cultured in Dulbecco’s modified Eagle’s medium (DMEM) (Thermo Fisher Scientific) supplemented with 10% heat-inactivated fetal bovine serum (FBS) (Gibco), Penicillin-Streptomycin (100 U/mL, 100 μg/mL (Sigma-Aldrich)), 1 mM sodium pyruvate (Sigma-Aldrich) and 10 ng/ml recombinant mouse IL-3 (Peprotech) and 10 ng/ml stem cell factor (SCF; Peprotech). Cells that were at least four weeks old were analysed for the expression of c-kit using an anti-CD117 antibody (2B8)-APC (BD Biosciences, # 553356) and FcɛRI (using an anti-FcɛRI antibody (MAR-1)-PE (Thermo Fisher scientific, #12-5898-82)) with a two laser Accuri instrument (BD Biosciences, San Jose, CA) and used for further experiments. The anti-mouse antibodies used above are described as target (clone)-fluorochrome conjugate, vendor, #catalog number. Peritoneal cell-derived MCs (PCMCs) were cultured as described [23, 50]. Immortalized Human Mast Cell Line (LUVA) was obtained from Kerafast (#EG1701-FP) and cultured in complete StemPro™-34 SFM (Thermo Fisher Scientific), supplemented with 2 mM L-glutamine or 1× GlutaMAX™ (Thermo Fisher Scientific) and penicillin–streptomycin in the presence of IL-3, SCF, and IL-6 (Peprotech). The cells were subcultured every 3–4 days. Primary human lung fibroblasts (HLFs) (PCS-201-013) and primary human lung smooth muscle cells (HLSMCs)(PCS-130-010) were obtained from American Type Culture Collection (ATCC, Manassas, VA) and cultured following the manufacturer’s instructions. Primary human small airway epithelial cells (HSAECs) (PCS-301-010) were obtained from ATCC and cultured in Airway Epithelial Cell Basal Medium (ATCC) containing a Bronchial Epithelial Cell Growth Kit (ATCC) and penicillin-streptomycin (100 U/mL, 100 μg/mL). Triple-negative human breast cancer cell line Hs578T was obtained from ATCC (HTB-126) and cultured at 37 °C in 5% CO_2_ in DMEM (Invitrogen) supplemented with 10% FBS, penicillin-streptomycin (50 μg/ml, 60 μg/ml), and 2 mM L-glutamine (Sigma-Aldrich). The cells were subcultured after reaching 90-100% confluency.

### Preparation of peripheral blood granulocytes

Eosinophil and neutrophil granulocytes were isolated from peripheral blood of healthy donors by Percoll gradient centrifugation as described [51]. Informed consent was obtained from all blood donors and the use of the blood samples for the present project was approved by the Uppsala Regional Ethical Review Board (Dnr 2020-05080).

### Nasal polyp tissue

Nasal polyps were obtained from participants undergoing Functional Endoscopic Sinus Surgery at Otorhinolaryngology and Head and Neck Surgery clinic, Uppsala University Hospital. The study was approved by the Uppsala Regional Ethical Review Board (Dnr 2022-06097-02) and written informed consent was provided by all participants. The tissue was collected immediately after surgical excision, transferred into ice-cold saline buffer, and stored on ice until used in experiments. Patients with polyps included both asthma and non-asthma subjects. Single cell suspensions from nasal polyps were obtained using a previously published protocol [52] with some minor modifications. Briefly, nasal polyp tissue was washed twice with PBS and cut into small pieces using scalpel blades and curved scissors. These pieces were further washed with PBS through a 70-μm filter to remove residual red blood cells (RBCs) and enzymatically digested for 30 min in digestion medium containing 2.8 mg/ml collagenase type IV (Sigma) and 0.03 mg/ml DNAse I (PanReac Applichem) in Roswell Park Memorial Institute (RPMI) 1640 medium (Gibco) supplemented with 10% FBS, Penicillin-Streptomycin (100 U/mL, 100 μg/mL) and 2 mM L-glutamine (complete RPMI) at 37 °C with stirring. After 30 min incubation, samples were mechanically disrupted using a syringe with 16 G needle for five times and incubated with the digestion medium for another 30 min. The enzymatic process was terminated by adding ice-cold complete RPMI. Next, samples were filtered through 40-μm and 70-μm cell strainers and centrifuged at 400xrcf (4 °C) for 10 min. RBCs were lysed using ACK buffer (Thermo Fisher Scientific) for 5 min on ice and samples were washed with PBS 2% FBS. Cells were further resuspended in PBS 2% FBS. Cell numbers and viability was determined using trypan blue (Thermo Fisher Scientific) exclusion and quantified by an automated cell counter (Countess TMII FL, Life Technologies) and/or hemocytometer. Extracted nasal polyp cells were resuspended in complete RPMI and subsequently incubated with PBS and/or TFP (10 μΜ) for 19 h in a humidified 37 °C incubator with 5% CO_2_, followed by flow cytometry analysis.

### Incubation of BMMCs with TFP and inhibitors

BMMCs were seeded in a 96-well plate in DMEM supplemented with 10% FBS, Penicillin-Streptomycin (100 U/mL, 100 μg/mL), and 1 mM sodium pyruvate. TFP was added to the cells at different concentrations and time periods and the plates were incubated at 37 °C in 5% CO_2_ in a humidified atmosphere. Cytotoxicity was assessed by flow cytometry. To investigate the effect of various protease inhibitors in response to TFP-induced cell death, BMMCs were pretreated with either the serine protease inhibitor Pefabloc SC (0.1 mM), the aspartic acid protease inhibitor pepstatin A (50 μΜ) or the cysteine protease inhibitor E64d (20 μΜ) for 30 min, followed by incubation with TFP. Cell death was assessed after 24 h. Caspase activation was evaluated by staining the cells with Caspase 3/7 Green Detection Reagent (2 μM) (Invitrogen). To this end, flow cytometric analysis of induced FITC fluorescence was performed. BMMCs treated with LLME (500 μΜ) for 1 h were included as positive control for caspase 3/7 activation [31]. To evaluate the enzymatic activity of caspase 3/7 inhibitor Z-DEVD-FMK, BMMCs were preincubated with Z-DEVD-FMK (20 μΜ) for 30 min followed by incubation with LLME (500 μΜ) for 1 h and measurement of caspase 3/7 activation. To delineate whether granule acidity mediates TFP-induced cell death, BMMCs were preincubated with the V-ATPase inhibitor bafilomycin A1 (20 nM) (Baf A1) followed by treatment with TFP. Caspase and granzyme B (GzmB) contribution to TFP-induced cell death was assessed by pretreating the cells with the pan-caspase inhibitor Z-VAD-FMK (20 μM) and Z-DEVD-FMK (20 μΜ) or the GzmB inhibitor Z-AAD-CMK (1, 10 μΜ), respectively, for 30 min followed by TFP treatment. When appropriate, the enzymatic activity of protease inhibitors and Z-AAD-CMK was tested using commercially available kits and/or fluorogenic substrates (Fig. S1).

### Cysteine cathepsin-like activity measurement

E64d inhibitor activity was measured using the chromogenic peptide substrate Z-Phe-Arg-AMC (Bachem) using a previously published protocol [31] with some minor modifications. Briefly, Hs578T cells were left untreated or treated with E64d (20 μΜ) and/or Pefabloc SC (0.1 mM) for 30 min at 37 °C. The cells were then collected in Eppendorf tubes and centrifuged at 1.200×rpm (4 °C) for 8 min. The cell pellet was washed with ice-cold PBS, centrifuged at 1.200×rpm (4 °C) for 8 min and lysed in 50 μL of lysis buffer (150 mM NaCl, 20 mM Tris-HCl, pH 7.2, 1% (v/v) Triton X-100). After 15 min incubation on ice, the supernatant was removed by centrifugation at 10.000×rcf (4 °C) for 1 min. Twenty μL lysate from each sample was then mixed with 20 μL H_2_O and 50 μL PBS containing 1 mM EDTA and 1 mM dithiothreitol (DTT) (pH 6.0), and transferred in triplicates into individual wells of a 96-well flat-bottomed plate. Samples were incubated for 15 min at 37 °C. Next, 10 μL of 200 μM Z-Phe-Arg-AMC was added followed by incubation for 30 min at 37 °C. Fluorescence was then measured with a TECAN Infinite M200 plate reader at an excitation wavelength of 390 nm and an emission wavelength of 460 nm.

### Cathepsin D activity assay

Pepstatin A (PesA) inhibitor activity was measured in cell lysates using a fluorometric cathepsin D (CTSD) activity assay kit (Abcam) according to manufacturer’s instructions. Briefly, BMMCs (1 × 10^6^) were incubated with or without PesA (50 μΜ) for 30 min at 37 °C. The cells were collected in Eppendorf tubes and centrifuged at 400×rcf (4 °C) for 5 min. The cell pellet was then washed with ice-cold PBS, centrifuged at 400×rcf (4 °C) for 4 min and lysed in 200 μL of chilled CD Lysis Buffer. After 10 min incubation on ice, samples were centrifuged at 10.000xrcf (4 °C) for 2 min. Equal amounts of sample were loaded in triplicates and CTSD substrate was added to the reaction. Plates were incubated for 1.5 h at 37 °C, protected from light. Fluorescence was then measured with a TECAN Infinite M200 plate reader at an excitation wavelength of 328 nm and an emission wavelength of 460 nm.

### Granzyme B activity measurement

Z-AAD-CMK activity was determined by measuring GzmB activity according to previously described protocols [53] with some modifications. Briefly, BMMCs were incubated with or without Z-AAD-CMK (1, 10 μΜ) for 30 min at 37 °C. Cell lysates were prepared as described above using the lysis buffer (150 mM NaCl, 20 mM Tris-HCl, pH 7.2, 1% (v/v) Triton X-100). Next, 5 μL from the resulting lysates (corresponding to 100.000 cells) were transferred in triplicates into individual wells of a 96-well flat-bottomed plates and mixed with 95 μL of a reaction buffer (50 mM Hepes, pH 7.5, 10% (w/v) sucrose, 0.05% (w/v) CHAPS, and 5 mM DTT) and 10 μL of N-Ac-IEPD-pNA (200 μM, dissolved in Me_2_SO). Plates were incubated at 37 °C, protected from light. Absorbance was then measured using a microplate reader (TECAN Infinite M200) at 405 nm.

### Flow cytometry

The effect of TFP on cell viability was assessed using Annexin V (AnnV) (BD Biosciences) and DRAQ7 (Abcam) staining. Viable cells were identified as double negative for Annexin V and DRAQ7, apoptotic cells were single positive for Annexin V and necrotic/late apoptotic cells were double positive for Annexin V and DRAQ7. BMMCs were identified based on their forward scatter area (*FSC-A*) and side scatter area (*SSC-A*) properties (Fig. S2). Granulocytes were gated by their forward- and side scatter properties. Additionally, eosinophils were identified as CD15^+^ CD193^+^(CCR3^+^), with lower fluorescence intensity of CD15 than in neutrophils. The anti-human antibodies used are described as target (clone)-fluorochrome conjugate, vendor, #catalog number: CD15 (W6D3)-PE (BD Biosciences, #562371), CD193 (5E8)-APC (Biolegend, #310707). The cells were analysed with a BD Accuri C6 plus flow cytometer. For nasal polyp tissue, single-cell suspensions were incubated with the following anti-human antibodies described as target (clone)-fluorochrome conjugate, vendor, #catalog number: CD45 (HI30)-eFluor™ 506 (Invitrogen, #50-112-4954), CD4 (RPA-T4)-BV421 (BD Biosciences, #562424), CD8 (RPA-T8)-BV421 (BD Biosciences, #562428), CD19 (HIB19)-BV421 (BD Biosciences, #562440), CD14 (M5E2)-FITC (BD Pharmingen, #557153), CD117 (104D2)-APC (Invitrogen, #17-1178-42), FcεRI (AER-37)-PE (BD Pharmingen, #556607). Single cell suspensions were stained with the antibody mix and LIVE/DEAD™ Fixable Near IR Dead cell stain kit (Invitrogen) for 30 min at 4 °C in the dark. After washing twice in FACS buffer (PBS with 0.1% BSA and 0.05% NaN_3_), flow cytometry analysis was performed on a Beckman Coulter Cytoflex S cytometer, and the data analyzed using FlowJo software (Tree Star Inc., Ashland, OR). Nasal polyp MCs, CD14^+^ monocytes, T- & B lymphocytes were gated as shown in Fig. S3Α.

### Measurement of granule and cytosolic pH

Intragranular pH alterations were assessed using LysoSensor Green (LSG) (Thermo Fisher Scientific). LSG is a pH indicator that accumulates in acidic compartments due to protonation. Cytosolic pH was measured using a Fluorometric Intracellular pH Assay Kit BCFL-AM (Sigma-Aldrich) according to the manufacturer’s instructions. This pH probe produces high fluorescence under basic conditions. BMMCs were pretreated with TFP or bafilomycin A1 (20 nM), followed by incubation with either probe. Fluorescence was monitored either by fluorescence microscopy (Nikon ECLIPSE 90i) equipped with NIS-Elements software (Nikon) or flow cytometry using an Accuri flow cytometer; a FITC filter was used to measure granule acidity and cytosolic pH. Data analysis was performed using the FlowJo software.

### Cytosolic extract preparation

Cytosolic extracts were prepared using digitonin extraction as previously described [31]. Supernatants (cytosolic extracts) were transferred to new Eppendorf tubes and protein concentration was determined using the Pierce™ BCA Protein Assay kit (Thermo Fisher Scientific). Samples were frozen until further use.

### Western blot analysis

Equal protein concentrations of cytosolic extracts (55 μg) were used for Western blot analysis of mouse tryptase (Mcpt6) using an anti-Mcpt6 antiserum (raised in rabbits; in house). Membranes were incubated with primary antibodies overnight and secondary antibody incubation was performed for 1 h. Images were acquired by using the ChemoDoc MP Imaging System (Bio-Rad). To verify consistency of protein concentrations between samples, replicate aliquots were run on a separate SDS-PAGE gel and stained with InstantBlue Coomassie Protein Stain (Expedeon) (Figure S4).

### DNA degradation

BMMCs previously treated with TFP and/or bafilomycin A1 were rinsed with ice-cold PBS and lysed (PBS, 0.2% Nonidet P-40, 0.4 mM EDTA, 12 μM pepstatin A, cOmplete™ EDTA-free Protease Inhibitor Cocktail (Roche)) for 14 min at 4 °C. Cells were then centrifuged at 12.000× rpm (4 °C) for 20 min and the supernatant was digested with 0.1 mg/ml RNAse for 1 h at 37 °C. The supernatant was further incubated with proteinase K (20 μg/ml) under the same conditions. Nucleic acids were precipitated by addition of 2-propanol, followed by incubation overnight at –20 °C. The samples were centrifuged at 12.000×rcf (4 °C) for 15 min and the pellets were incubated with ice-cold 70% ethanol for 20 min on ice. Next, samples were centrifuged at 12.000×rcf (4 °C) for 10 min and pellets air-dried for 30 min. Next, samples were resuspended in Tris acetate EDTA (TAE) buffer supplemented with sample buffer (0.25% bromphenol blue, 30% glycerol). Samples were then subjected to electrophoresis on 2% agarose gels at 100 V for 45 min, and for a further 10 min at 120 V. Gels were visualized under UV light after staining with GelRed Nucleic Acid Gel Stain (Biotium).

### Staining of BMMCs

BMMCs were centrifuged onto cytospin glass slides (Cytospin, Shandon Southern Instruments, Sewickley, P) and air-dried overnight. The cells were stained with toluidine blue (Sigma-Aldrich) following a standard protocol [49]. Cell morphology was assessed using light microscopy.

### Transmission electron microscopy (TEM)

BMMCs were fixed in 2.5% Glutaraldehyde (Ted Pella) and 1% Formaldehyde (Merck) in Phosphate Buffer (PB) (0.1 M, pH 7.4) and stored at 4 °C until further processing. Next, cells were rinsed with PB for 10 min followed by post-fixation in 1% osmium tetroxide (Agar Scientific) for 1 h. Cells were dehydrated in a graded series of ethanol (50%, 70%, 95% and 99.9%) for 10 min and propylene oxide (Agar Scientific) for 5 min. Subsequently, the cells were placed in a mixture of Epon Resin (Agar Scientific) and propylene oxide (1:1) for 1 h, followed by 100% resin and left overnight. Newly prepared Epon resin was added and left for 1–2 h. Next, the Epon resin was polymerized at 60 °C for 48 h. EM UC7 Ultramicrotome (Leica) was used to cut Ultrathin sections (60–70 nm). The sections were placed on a grid and contrasted with 5% uranyl acetate and 3% Reynold’s lead citrate (Science Services). Analysis was performed on a Tecnai™ G2 Spirit BioTwin transmission electron microscope (Thermo Fisher Scientific/FEI) at 80 kV equipped with an ORIUS SC200 CCD camera and Gatan Digital Micrograph software (both from Gatan Inc.).

### Statistical analysis and graphs

Data were analysed using the GraphPad Prism 9.3 statistics and graphing software (GraphPad Software). If not indicated otherwise, statistical differences between three or more groups were assessed using one-way ANOVA with Dunnett’s multiple comparison test or when appropriate, non-parametric Kruskal-Wallis test with Dunn’s multiple comparison tests. Comparisons were performed relative to either untreated (control) or TFP-treated MCs as indicated in the figure legends. For comparisons between two groups, unpaired Student’s *t*-test was used. The results shown are from individual experiments, representative of at least 2 experiments. Statistical significance was set at *P* < 0.05.

## Supplementary information

Uncropped Western blot inage

Figure S1

Figure S2

Figure S3

Figure S4

Figure S5

## Data Availability

All data generated and analysed during this study are included in this article. Further details or raw data are available from the corresponding authors upon reasonable request.
