# Supplementary figures and images for: Trifluoperazine causes mast cell apoptosis through a secretory granule-mediated pathway
Source: Cell Death Discov. 2026 Apr 22;12:185. doi: 10.1038/s41420-026-03122-x (PMC13103083; doi:10.1038/s41420-026-03122-x)

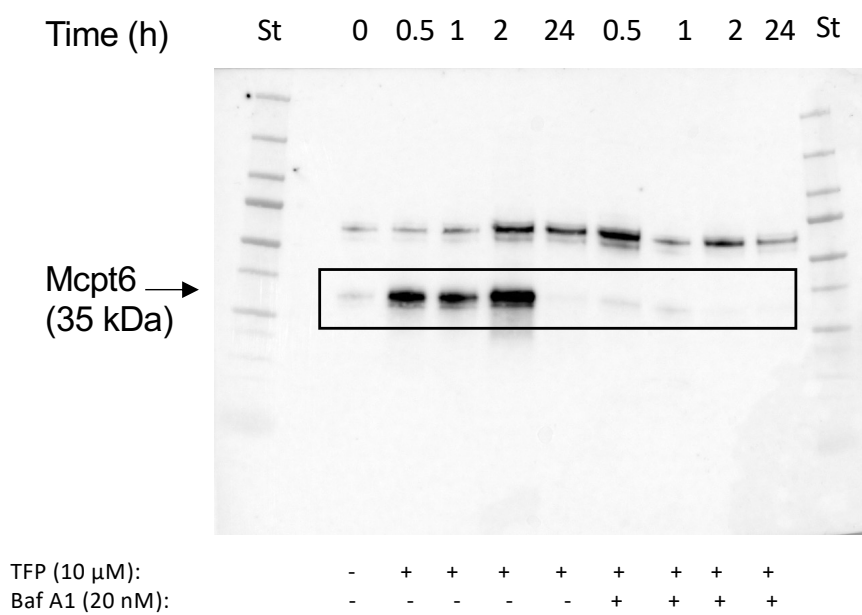

Uncropped imagel, Western blot

Supplement: Supplementary file 1 — Uncropped Western blot inage [file 41420_2026_3122_MOESM1_ESM.pdf]
