# Supplementary material for: Trifluoperazine causes mast cell apoptosis through a secretory granule-mediated pathway
Source: Cell Death Discov. 2026 Apr 22;12:185. doi: 10.1038/s41420-026-03122-x (PMC13103083; doi:10.1038/s41420-026-03122-x)
Supplement: Supplementary file 2 — Figure S1 [file 41420_2026_3122_MOESM2_ESM.pdf]

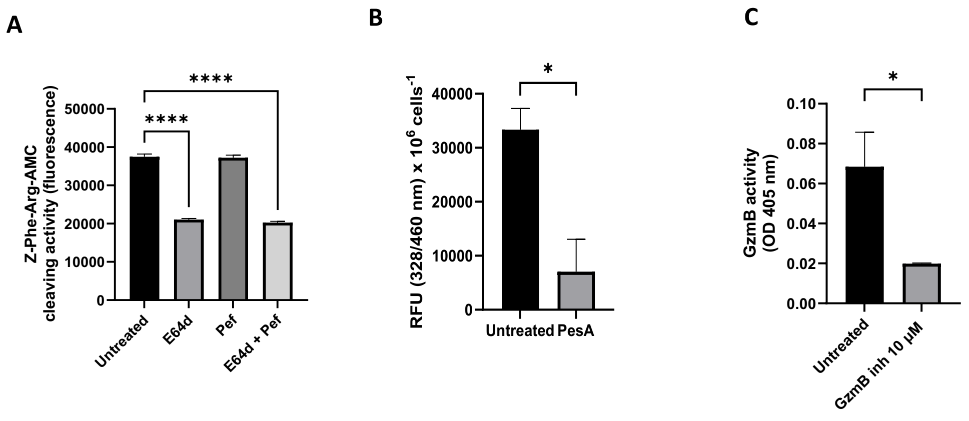

Figure S1. Evaluation of inhibitors used to assess cysteine protease-, aspartic acid protease- and granzyme B (GzmB) activity. (A) Residual Z-Phe-Arg-AMC cleaving activity was measured after incubation of Hs578T cell lysates treated with or without E64d (20 µM) and/or Pefabloc SC (Pef) (0.1 mM) for 30 min. Incubation with Pef was included to rule out cleavage by serine proteases acting at Arg residues of the substrate. n=3 from one individual experiment, representative of three independent experiments (One-way ANOVA). (B) Cathepsin D assay activity was performed on BMMC lysates incubated with or without pepstatin A (PesA) (50 µM) for 30 min. Data is expressed as relative fluorescence units (RFU) per million cells. n=3 from one individual experiment, representative of three independent experiments (two-tailed unpaired t-test). (C) BMMC lysates treated with or without GzmB inh Z-AAD-CMK (10 µM) 30 min were assayed for granzyme B activity with N-Ac-IEPD-pNA. n=3 from one individual experiment, representative of three independent experiments (two-tailed unpaired t-test). Untreated (control) cell lysates were used for statistical comparisons to all other groups in all figures. The bar charts show mean values + SEM. *P < 0.05; ****P < 0.0001.
