# Supplementary material for: Trifluoperazine causes mast cell apoptosis through a secretory granule-mediated pathway
Source: Cell Death Discov. 2026 Apr 22;12:185. doi: 10.1038/s41420-026-03122-x (PMC13103083; doi:10.1038/s41420-026-03122-x)
Supplement: Supplementary file 3 — Figure S2 [file 41420_2026_3122_MOESM3_ESM.pdf]

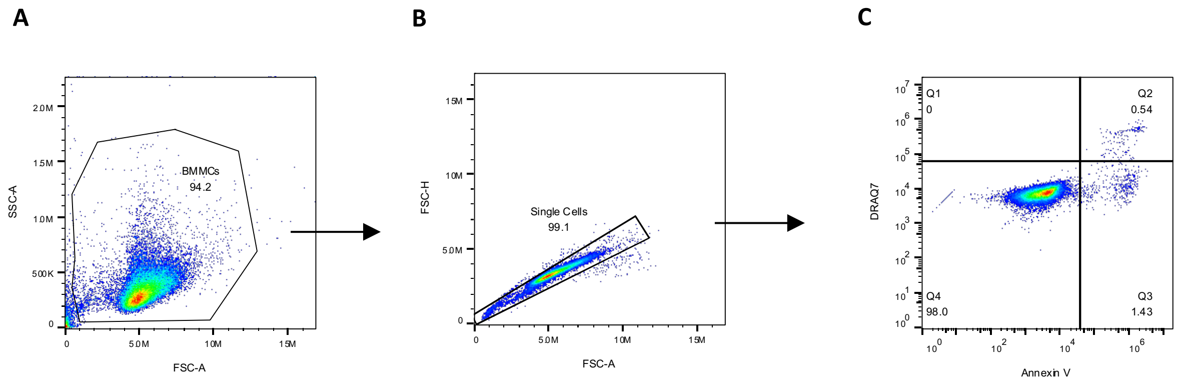

Figure S2. General gating strategy for BMMCs. (A) Identification of BMMCs based on their forward scatter area (*FSC-A*) and side scatter area (*SSC-A*) properties and exclusion of debris. (B) Exclusion of doublets. (C) Differentiation of cell populations regarding viability**.**
