# Supplementary material for: Trifluoperazine causes mast cell apoptosis through a secretory granule-mediated pathway
Source: Cell Death Discov. 2026 Apr 22;12:185. doi: 10.1038/s41420-026-03122-x (PMC13103083; doi:10.1038/s41420-026-03122-x)
Supplement: Supplementary file 4 — Figure S3 [file 41420_2026_3122_MOESM4_ESM.pdf]

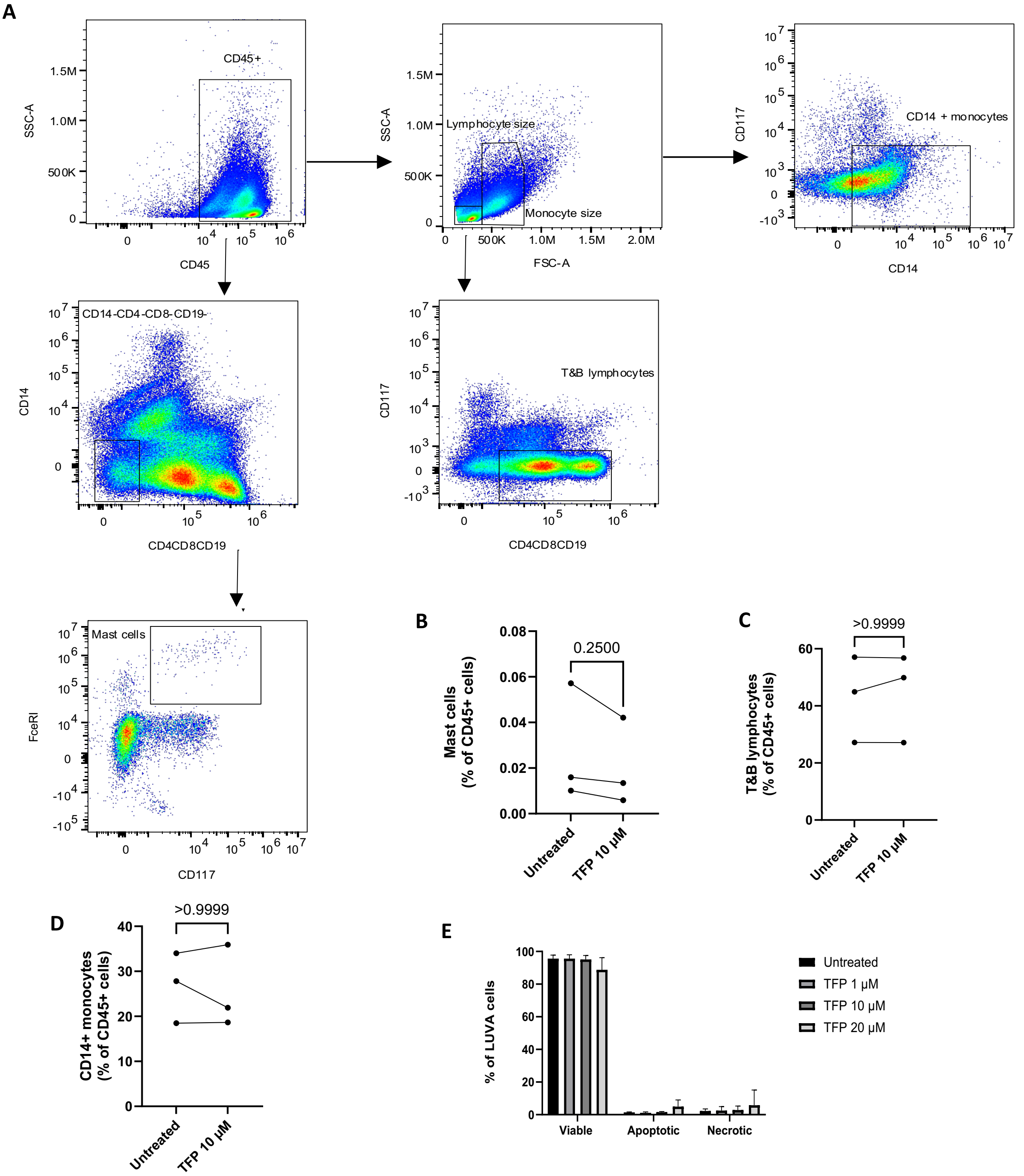

Figure S3. Effect of trifluoperazine (TFP) on LUVA cells and on the frequency of immune cells in human nasal polyps. Mixed human nasal polyp cells were prepared by mechanical and enzymatic dissociation and treated with or without TFP (10 µM) for 19 h. (A) Representative gating strategy used to determine the frequency of nasal polyp mast cells ($CD45^+$ $CD4^-$ $CD8^-$ $CD19^-$ $CD14^-$ c-kit^+ $Fc\varepsilon RI^+$), T&B lymphocytes ($CD45^+$ FSC^{low} SSC^{low} c-kit^- $CD4^+$ $CD8^+$ $CD19^+$) and $CD14^+$ monocytes/macrophages ($CD45^+$ FSC^{high} SSC^{low} c-kit^- $CD14^+$). (B-D) Frequency of nasal polyp mast cells, T&B lymphocytes and $CD14^+$ monocytes in response to treatment with or without TFP. n=3 from three independent experiments / three individual donors (Wilcoxon matched-pairs signed rank-test). (E) Non-transformed MC line (LUVA) was treated with TFP at the indicated concentrations for 24h. Cell viability was assessed by staining the cells with AnnV and DRAQ7. Viable cells, $AnnV^-$ $DRAQ7^-$; apoptotic cells, $AnnV^+$ $DRAQ7^-$; necrotic/late apoptotic cells, $AnnV^+$ $DRAQ7^+$. n=4 from four independent experiments (Kruskal-Wallis). ns, not significant. Untreated (control) cells were used for statistical comparisons to all other groups in all graphs. The bar charts show median + interquartile range.
