# Supplementary material for: Trifluoperazine causes mast cell apoptosis through a secretory granule-mediated pathway
Source: Cell Death Discov. 2026 Apr 22;12:185. doi: 10.1038/s41420-026-03122-x (PMC13103083; doi:10.1038/s41420-026-03122-x)
Supplement: Supplementary file 5 — Figure S4 [file 41420_2026_3122_MOESM5_ESM.pdf]

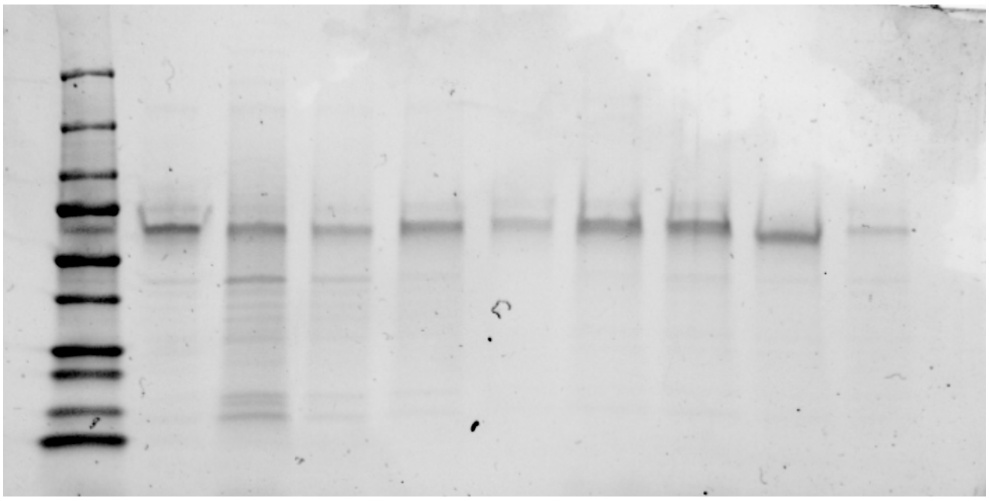

Figure S4. Proteins from cytosolic extracts were separated by SDS-PAGE and stained with InstantBlue. BMMCs were preincubated with or without bafilomycin A1 (Baf) (20 nM) for 4h followed by treatment with TFP (10 µM) for 20h. Cytosolic extracts were prepared and equal protein concentrations (55 µg) were loaded into SDS-PAGE. Staining of the gel with InstantBlue Coomassie Protein Stain was performed for 2h and consistency of protein samples (replicates) was assessed.
