# Supplementary material for: Trifluoperazine causes mast cell apoptosis through a secretory granule-mediated pathway
Source: Cell Death Discov. 2026 Apr 22;12:185. doi: 10.1038/s41420-026-03122-x (PMC13103083; doi:10.1038/s41420-026-03122-x)
Supplement: Supplementary file 6 — Figure S5 [file 41420_2026_3122_MOESM6_ESM.pdf]

A

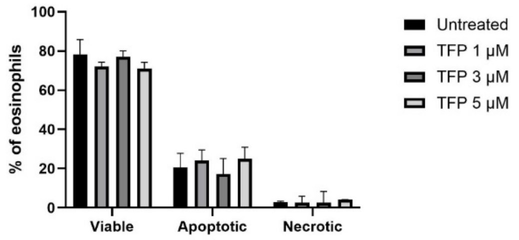

B

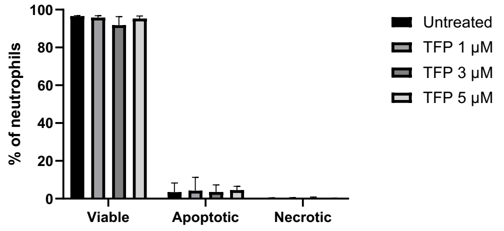

Figure S5. Trifluoperazine (TFP) at low concentrations does not affect viability of human peripheral blood eosinophils or neutrophils. (A) Eosinophils and (B) neutrophils were treated with TFP at the indicated concentrations for 2h. Cell viability was assessed by staining the cells with AnnV and DRAQ7. Viable cells, $AnnV^{-}$ $DRAQ7^{-}$; apoptotic cells, $AnnV^{+}$ $DRAQ7^{-}$; necrotic/late apoptotic cells, $AnnV^{+}$ $DRAQ7^{+}$. n=3 from three independent experiments/three individual donors (Friedman test with Dunn's multiple comparison test). Untreated (control) cells were used for statistical comparisons to all other groups in all graphs. The bar charts show median+ interquartile range.
